# Pain Medication in the Elderly Patient

**DOI:** 10.1192/j.eurpsy.2022.154

**Published:** 2022-09-01

**Authors:** F. Riese

**Affiliations:** University of Zurich, University Research Priority Program „dynamics Of Healthy Aging“, Zurich, Switzerland

**Keywords:** opioids, nsaid, geriatrics, old age psychiatry

## Abstract

This presentation highlights core pharmacological aspects of opioid and non-opioid pain medications in the elderly patient. Specifically, it covers pharmakinetics, pharmacodynamics and drug-drug interactions of select pain medications. The presentation aims to promote safe use of pain medications in the elderly.

**Disclosure:**

No significant relationships.

